# Resting-State Functional Brain Connectivity and Neurodevelopmental Profiles in 3-Year-Olds with Congenital Heart Disease

**DOI:** 10.3390/biomedicines14102226

**Published:** 2026-10-01

**Authors:** Charlotte Savard, Julie Tremblay, Sarah Provost, Natacha Paquette, Sarah Kessouri, Kassandra Roger, Solène Fourdain, Amélie Doussau, Phetsamone Vannasing, Marie-Noëlle Simard, Anne Gallagher

**Affiliations:** 1 Azrieli Research Center, Sainte-Justine University Hospital, Montréal, QC H3T 1C5, Canada; charlotte.savard@umontreal.ca (C.S.); julie.tremblay2.hsj@ssss.gouv.qc.ca (J.T.); sarah.provost@umontreal.ca (S.P.); natacha.paquette.hsj@ssss.gouv.qc.ca (N.P.); sarah.kessouri.hsj@ssss.gouv.qc.ca (S.K.); kassandra.roger@umontreal.ca (K.R.); solene.fourdain@umontreal.ca (S.F.); pvannasing@gmail.com (P.V.); marie-noelle.simard@umontreal.ca (M.-N.S.); 2 Department of Psychology, Université de Montréal, Montréal, QC H3C 3J7, Canada; 3 Clinique d’Investigation Neurocardiaque (CINC), CHU Sainte-Justine University Hospital, Montréal, QC H3T 1C5, Canada; amelie.doussau.hsj@ssss.gouv.qc.ca; 4 School of Rehabilitation, Université de Montréal, Montréal, QC H3C 3J7, Canada

**Keywords:** congenital heart disease, resting-state functional brain connectivity, neurodevelopment, intellectual abilities, language abilities, motor skills, executive functions

## Abstract

**Background/Objectives**: Congenital heart disease (CHD) is associated with altered brain development and an increased risk of neurodevelopmental difficulties, particularly in more severe forms of the condition. Although alterations in functional brain connectivity have been reported before and after the first corrective surgery in early infancy, as well as in adulthood, little is known about their presentation in early childhood and their association with neurodevelopmental outcomes. This study aimed to characterize resting-state functional connectivity in 3-year-old children with moderate-to-severe CHD and to examine its associations with neurodevelopmental outcomes, including cognitive, language, and motor abilities. **Methods:** Twenty-five 3-year-old children with moderate-to-severe CHD and 28 age-matched healthy controls completed resting-state functional near-infrared spectroscopy (fNIRS) analysis and a standardized neurodevelopmental assessment. Connectivity was compared across networks using a repeated-measures analysis of covariance (ANCOVA), then within each network separately, controlling for maternal socioeconomic status and with sex as a factor. Associations with neurodevelopmental measures were examined using hierarchical linear regressions adjusted for group, sex, and socioeconomic status, with connectivity-by-group interactions tested. **Results:** Children with moderate-to-severe CHD showed lower resting-state functional connectivity than controls specifically within the right fronto-temporal network, with no group differences in the four remaining networks, as well as poorer language performance. Greater inter-hemispheric functional connectivity was associated with fewer executive function difficulties and better overall motor performance across participants, and greater frontal connectivity with better motor coordination, with no significant differences in these associations between groups. **Conclusions:** Preschool-aged children with CHD show regionally circumscribed alterations in functional connectivity and language vulnerabilities, while the associations between connectivity and executive and motor functioning appear to extend across both CHD and typically developing children.

## 1. Introduction

Congenital heart disease (CHD) refers to structural anomalies of the heart and surrounding vessels and is the most common congenital anomaly in newborns [1,2]. CHD arises during fetal development and, although its exact cause remains unclear, it is attributed to a variety of genetic and environmental factors [3,4,5]. CHD encompasses a wide spectrum of severity, with more complex forms involving significant disruptions in cardiac development that, in turn, may compromise cerebral oxygenation and nutrient delivery during the third trimester of pregnancy, a critical period for brain maturation [6,7,8]. In addition, genetic influences, early cardiac surgery, and repeated hospitalizations during infancy may further contribute to altered brain development [5,9,10]. Together, these factors may adversely affect neurodevelopment and the maturation of cerebral networks [11,12].

Approximately 50% of children with moderate-to-severe CHD experience difficulties across developmental domains [6,13]. Moderate CHD encompasses conditions requiring specialized care, while severe CHD generally includes conditions associated with serious illness in the newborn period or early infancy and requiring more intensive care [2]. Although these impairments are often subtle, they are widespread and may affect motor, language, cognitive, behavioral, social, and psycho-affective functioning [7,14,15,16]. While some difficulties can be identified in early childhood, many remain undetected until later developmental stages, when increasing environmental and academic demands exacerbate these challenges [17,18,19,20]. Consequently, moderate-to-severe CHD has been associated with adverse long-term functional outcomes, including lower academic achievement, poorer psychosocial adjustment, and reduced quality of life in adulthood [17,21,22].

Structural brain abnormalities are frequent in children with moderate-to-severe CHD, including white matter injury, reduced brain volume, ventricular enlargement, and altered cortical folding [8,11,23,24]. Beyond these structural alterations, growing evidence suggests that atypical cerebral connectivity may be a key mechanism underlying neurodevelopmental outcomes in this population [25,26]. Altered structural brain organization, including disruptions of the structural connectome, has been associated with poorer developmental outcomes in children with CHD and other at-risk populations [26,27,28,29,30,31].

Brain connectivity can also be examined from a functional perspective by looking at brain regions that show synchronous activity, at rest or during a task, rather than physical connections between them [31]. A growing body of neuroimaging research has characterized functional brain organization in both typically developing children and children with CHD during cognitive tasks [28,32,33,34]. Resting-state functional connectivity further extends this work by providing insight into the spontaneous organization of functional brain networks in the absence of task demands [28,29]. Altered functional connectivity has been observed in neonates, infants, and adolescents with CHD [28,30,35]. Similar to structural connectivity, these functional alterations may result from brain injury but may also reflect broader brain dysmaturation associated with moderate-to-severe CHD, suggesting an atypical developmental trajectory [25,30]. Importantly, early differences in functional connectivity have been associated with poorer neurodevelopmental outcomes [28,36,37].

Despite growing interest in brain connectivity in individuals with CHD, to our knowledge, there are very few studies looking at functional brain organization during early childhood, a developmental period associated with rapid brain network maturation.

Therefore, the present study aims to characterize resting-state functional connectivity alterations in 3-year-old children with moderate-to-severe CHD. The second objective is to investigate whether these alterations are associated with distinct neurodevelopmental domains, including cognitive, language, and motor abilities.

## 2. Materials and Methods

### 2.1. Participants

In this prospective study, 25 children with moderate-to-severe CHD were recruited between 2019 and 2023 at the neurocardiac developmental clinic at Sainte-Justine University Hospital, all being 3 years of age (CHD group, 41–47 months; M = 43.60, SD = 1.50). CHD severity was classified according to Hoffman and Kaplan [2], with moderate and severe lesions. All children had undergone at least one open-heart surgery within the first three years of life. Exclusion criteria included genetic syndromes (e.g., Down Syndrome), preterm birth (<37 weeks of gestational age), low birth weight (<2500 g), and any major neurological or medical conditions significantly affecting neurodevelopment. Medical records were used to validate eligibility. Twenty-eight age-matched healthy children were also recruited (control group) in the community using laboratory databases, online advertisements, and hospital-based recruitment strategies. Eligibility was assessed through a review of medical records, when available, or by using detailed questionnaires. For all participants (CHD and controls), demographic characteristics, including maternal occupation, were collected using questionnaires. Maternal socioeconomic status (SES) was then estimated using the Nam–Powers–Boyd occupational status scores adapted for the 2001 Canadian Census [38]. The study was approved by the Research Ethics Board of the Sainte-Justine University Hospital Center, and parents provided written consent prior to participation.

### 2.2. Procedure

Participation in this study required two visits to the laboratory. The first visit consisted of a 90-min developmental assessment conducted by a clinical neuropsychology graduate student (S.P.). During the second visit, participants completed a 75-min functional near-infrared spectroscopy (fNIRS) session that included a resting-state acquisition.

### 2.3. Resting-State Functional Near-Infrared Spectroscopy

The main objective of this study was to characterize resting-state functional connectivity in 3-year-old children with moderate-to-severe CHD. To this end, resting-state fNIRS data were acquired using a continuous-wave NIRScout system (NIRx Medical Technologies, Orlando, FL, USA) equipped with 16 sources and 24 detectors operating at wavelengths of 760 and 850 nm, sampled at 7.8 Hz. Optode placement followed the international 10-10 system [39], resulting in 62-source-detector channels, with source-detector distances ranging from 2.5 to 4 cm. Eight short-separation detectors positioned 8 mm from the standard emitter optodes were incorporated in the original fNIRS montage [40]. However, short-separation regression was not applied in the final preprocessing pipeline, as preliminary analyses indicated that incorporating the short-separation signals removed a substantial portion of the signal of interest (i.e., cerebral signal) rather than selectively capturing extracerebral contributions. Consequently, short-separation signals were not assigned to corresponding long-separation channels or included as nuisance regressors. Instead, global signal regression (GSR) was applied to reduce spatially widespread signal components, including those potentially related to systemic physiological contributions [41,42].

A flexible cap (EasyCap GmbH, Wörthsee-Etterschlag, Germany) holding the detectors and sources in place was positioned according to standard anatomical references. The montage covered frontal, temporal, and parietal regions. Probe localization was digitized on a head model using the Polaris stereotaxic system (Northern Digital Inc., Waterloo, ON, Canada) and Brainsight Frameless software (version 1.5; Rogue Research Inc., Montréal, QC, Canada). A 33–44-month-old child structural magnetic resonance imaging template [43] was used in conjunction with the 3DMTG interface from the LIONirs toolbox [44] to visualize probe locations.

Data acquisition took place in a dimly lit, soundproof room, with children seated comfortably either alone or on their parent’s lap, although children with CHD were more frequently seated on their parent’s lap (47.4%) than controls (18.5%). Cap installation was facilitated by showing a movie to increase cooperation. A senior fNIRS-electrophysiology technician (P.V.) supervised the installation, ensured appropriate optode positioning and signal quality, and monitored the child’s attention and behavior throughout the recording. A night-vision camera recorded the session to allow for artifact identification (e.g., movement, yawning). Participants completed a 12-min resting-state acquisition while passively viewing a black cross on a grey screen.

### 2.4. fNIRS Data Processing

Data preprocessing was performed using the LIONirs toolbox [44], implemented within SPM12 [45], running in MATLAB (version R2024b; The MathWorks, Inc., Natick, MA, USA). Raw light intensity data were first converted to optical density. Channel quality was assessed based on the presence of a visible cardiac pulse, a well-established physiological marker in fNIRS [44]. Channels not meeting this quality criterion were excluded. Exclusive rejection to address motion artifacts has been discouraged in the fNIRS literature, including in studies involving infants, as it reduces the number of valid trials and impacts the accuracy of the hemodynamic response function [46,47]. Therefore, visual inspection was performed to identify and correct motion artifacts using principal component analysis (PCA) and parallel factor analysis (PARAFAC) decomposition methods, based on abrupt amplitude changes and perturbations concurrent across multiple channels. PCA was used for relatively simple, spatially widespread artifacts, whereas PARAFAC was used for more complex, non-orthogonal, or spatially heterogeneous artifacts requiring separation across multiple dimensions, as was the case for most artifacts identified in the present dataset [48,49,50]. Components were removed iteratively until the artifact was no longer visually apparent. Although preprocessing was not blinded to diagnostic group, identical procedures were applied to all participants, and quality control was reviewed by a biomedical engineer (J.T.). Segments containing artifacts were processed using targeted decomposition algorithms to remove abnormal components while preserving underlying hemodynamic activity [44,50]. Offset adjustments were applied to ensure signal continuity.

The data were low-pass-filtered at 0.2 Hz using a fourth-order Butterworth digital filter with zero-phase forward and reverse filtering to reduce respiratory and cardiac confounds. Hemodynamic signals were derived using the modified Beer–Lambert law, converting optical density changes into relative concentrations of oxyhemoglobin (HbO) and deoxyhemoglobin (HbR) using an age-adapted differential pathlength factor [51]. HbO concentration changes were used for subsequent analyses, as they are more sensitive than HbR to hemodynamic changes associated with neural activity [52,53]. A sensitivity analysis using HbR signals was also conducted to assess the robustness of the findings and is reported in the Results.

After fNIRS data preprocessing, each participant’s recording was segmented into 200 randomly selected 30-s intervals as part of the bootstrap procedure [44,54]. Random sampling of multiple segments from resting-state recordings has previously been used to estimate functional connectivity in fNIRS [36]. Segment start points were randomly selected across the resting-state recording, and intervals were allowed to overlap. Excessively noisy segments were excluded. Each segment was detrended to remove very-low-frequency drift; detrending was applied rather than a high-pass filter to avoid the filtering artifacts that a hard cutoff can introduce in relatively short fNIRS resting-state recordings. Then, for each 30-s segment, functional connectivity was calculated as the Pearson correlation coefficient between each pair of channels.

To support reproducibility, the LIONirs toolbox used for data preprocessing and functional connectivity analyses is open-source and freely available online (https://github.com/JulieTremblay3/LIONirs, accessed on 18 September 2026).

### 2.5. Functional Networks of Interest

**Figure 1:**
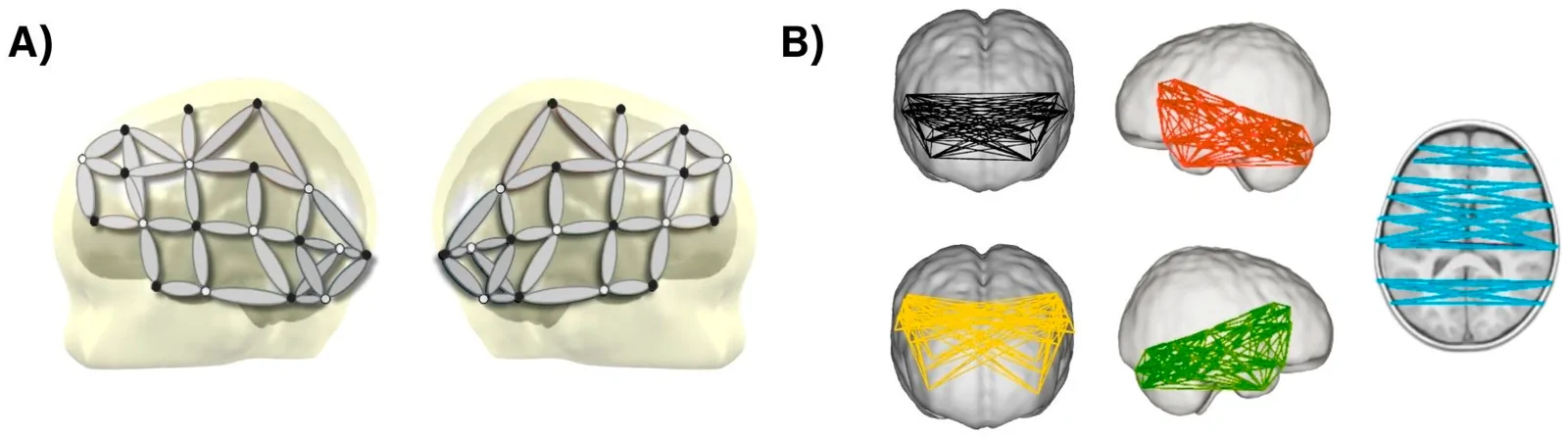
(**A**) Functional near-infrared spectroscopy (fNIRS) montage illustrating channel placement over frontal, temporal, and parietal cortical regions. Sources are shown as white circles with black outlines, and detectors as solid black circles. (**B**) Visualization of the five functional connectivity networks examined in the present study: frontal (black), fronto-parietal (yellow), left fronto-temporal (orange), right fronto-temporal (green), and inter-hemispheric (blue).

The fNIRS montage covered frontal, temporal, and parietal cortical regions (Figure 1A). Based on the early neurodevelopmental resting-state network literature, five functional networks were defined within these cortical areas: frontal, fronto-parietal, left fronto-temporal, right fronto-temporal, and inter-hemispheric networks. These networks encompass brain regions that play a central role in the development of cognitive, language, and motor abilities during the preschool years, all of which undergo rapid maturation during early childhood and are critical for neurodevelopmental outcomes [28,37,55,56,57]. To construct these networks, channels were first assigned to anatomically defined regions of interest (ROIs). Functional connectivity was then calculated for all pairwise channel connections, and connections linking the corresponding ROIs were grouped to form the five predefined functional networks: frontal (105 connections), left fronto-temporal (165 connections), right fronto-temporal (165 connections), fronto-parietal (56 connections), and inter-hemispheric (127 connections) (Figure 1B).

Connectivity estimates were averaged across segments for each channel pair and Fisher z-transformed, then averaged across all participants to yield a single group-level connectivity matrix. Within each ROI, channel pairs were ranked by absolute connectivity strength and the strongest 25% were retained. This mask was applied identically to every participant, such that all participants contributed the same channel pairs to each ROI. Participant-level ROI estimates were then obtained by averaging the signed connectivity values of the retained pairs.

Density thresholding of this kind is the most commonly applied edge-selection strategy in functional network analysis, with thresholds retaining the strongest 10–30% of connections representing standard practice [58], and has been used with resting-state fNIRS channel matrices [59]. The threshold was applied to the group-averaged matrix rather than to individual matrices, as resting-state fNIRS connectivity has been shown to be more reliable at the group than at the individual level, with channel-wise individual estimates in particular showing low reliability in the absence of optode co-registration; group-level analysis attenuates individual variability and the imperfect matching of probe placement across participants [60]. Because the group matrix pooled both diagnostic groups and was blind to neurodevelopmental outcomes, connection selection was independent of the effects subsequently examined.

No definitive criterion exists for selecting a single threshold value [61]; the 25% threshold was selected a priori and retained as it falls within the range typically reported in the literature. Furthermore, it was the lowest threshold that preserved data for the full sample, with lower thresholds producing missing values once ROIs were restricted to channel pairs common to all participants.

### 2.6. Neurodevelopmental Assessment

To examine the association between resting-state functional connectivity and neurodevelopmental functioning (Objective 2), all children underwent a standardized neurodevelopmental assessment. Global cognitive and language development was assessed using the French-Canadian version of the Wechsler Preschool and Primary Scale of Intelligence, Fourth Edition (WPPSI-IV), a standardized assessment of cognitive abilities in children aged 2 years 6 months to 7 years 7 months [62]. Participants completed five subtests assessing receptive vocabulary (Receptive Vocabulary), visuospatial reasoning (Block Design), acquired knowledge (Information), visual-spatial integration (Object Assembly), and expressive vocabulary (Picture Naming) (M = 10, SD = 3). These yielded two primary index scores, the Verbal Comprehension Index (VCI) and the Visual Spatial Index (VSI), as well as two ancillary index scores, the Vocabulary Acquisition Index (VAI) and the General Ability Index (GAI). Scaled scores (M = 10, SD = 3) were also calculated for each subtest and used in subsequent analyses. All index scores are standardized and validated for use in Canada, with a mean of 100 and a standard deviation of 15.

Executive functioning in everyday contexts was assessed using the Behavior Rating Inventory of Executive Function, preschool version (BRIEF-P) [63], which contains 4 indices: the Inhibitory Self-Control Index (ISCI), the Flexibility Index (FI), the Emergent Metacognition Index (EMI), and the Global Executive Composite (GEC). The questionnaire was completed by a primary caregiver. The BRIEF-P is a standardized measure designed for children aged 2 years to 5 years, 11 months. Raw scores are converted into standardized T scores, with higher scores reflecting greater parent-reported executive difficulties across all scales.

Motor development was assessed using the Peabody Developmental Motor Scales, second edition (PDMS-2) [64]. Age-standardized composite scores (M = 100, SD = 15) were obtained for the Fine Motor Quotient (FMQ), Gross Motor Quotient (GMQ), and Total Motor Quotient (TMQ) and used in subsequent analyses. Higher scores indicate better motor functioning. Visuomotor integration was measured using the Beery–Buktenica Developmental Test of Visual–Motor Integration, Sixth Edition (Beery-VMI) [65]. Age-standardized scaled scores (M = 10, SD = 3) were obtained for the Visual–Motor Integration (VMI) and Motor Coordination (MC) subtests and used in subsequent analyses. The Visual Perception subtest was not included because it was not directly relevant to the study objectives. Higher scores reflect better visual-motor integration abilities.

### 2.7. Statistical Analysis

Descriptive statistics were computed to characterize sociodemographic and clinical variables, including maternal SES. Group differences in sociodemographic variables were examined using independent-samples Student’s *t*-tests, with Welch’s correction applied when the assumption of homogeneity of variance was violated, and chi-square tests for categorical variables. Prior to the analyses, model assumptions were evaluated, and no substantial violations of these assumptions were observed. Sample sizes varied across analyses according to data availability for the measures and covariates included in each model; the number of participants included is therefore reported for each analysis.

Resting-state functional brain connectivity was estimated using Pearson correlation coefficients derived from HbO signals and transformed using Fisher’s r-to-z transformation prior to statistical analyses to stabilize variance and improve normality.

Differences in connectivity across networks were examined using a repeated-measures analysis of covariance (ANCOVA), with functional network (frontal, left and right fronto-temporal, fronto-parietal, and inter-hemispheric) as a within-subject factor, group (CHD, controls) and sex as between-subjects factors, and maternal SES as a covariate. Post hoc comparisons between networks were conducted using Bonferroni-adjusted tests.

Group differences were then examined within each network separately, using univariate ANCOVAs with group and sex as factors and maternal SES as a covariate. Networks were predefined, and analyses were therefore conducted for all five, with a Bonferroni-corrected threshold of α = 0.01 applied across the five comparisons. Bonferroni correction was used here given the small number of predefined comparisons, whereas false discovery rate (FDR) correction was applied to the larger families of neurodevelopmental tests described below.

Sex was included as a between-subjects factor because the CHD and control groups differed significantly in their sex distribution (see Table 1). Maternal SES was included as a covariate because it is a well-established predictor of neurodevelopmental outcomes in both children with CHD and the general population [66,67,68]. Given the small number of girls in the CHD group, a sensitivity analysis restricted to male participants was also conducted to evaluate the robustness of the group effect on functional connectivity.

Neurodevelopmental measures were grouped into three domains: cognition, language, and motor functioning. Each neurodevelopmental score was analyzed using a separate ANCOVA, with group and sex as fixed factors and maternal SES included as a covariate. FDR correction was applied separately within each of the three neurodevelopmental domains (cognitive, language, and motor), such that all outcome measures within a given domain constituted a single family of tests. Statistical significance was determined based on FDR-adjusted *p*-values, with a threshold of <0.05.

To examine associations between functional brain connectivity and neurodevelopmental measures, separate hierarchical linear regression models were performed for each functional network and neurodevelopmental measure. Diagnostic group, sex, and maternal SES were entered in the first step, followed by functional connectivity in the second step to assess its association with neurodevelopmental outcomes after adjustment for these covariates. A connectivity-by-group interaction term was entered in the third step to determine whether the association between functional connectivity and neurodevelopmental outcomes differed between groups. FDR correction was applied separately within each neurodevelopmental domain and separately for the connectivity main effects and connectivity-by-group interaction effects, with statistical significance set at α < 0.05.

Raw and FDR-adjusted *p*-values are reported in the main tables and in Supplementary Tables S1 and S3.

All statistical analyses were conducted using IBM SPSS Statistics (version 31.0; IBM Corp., Armonk, NY, USA). Figures were generated using IBM SPSS Statistics, MATLAB, and RStudio (version 2024.12.1+563; Posit Software, PBC, Boston, MA, USA), depending on the type of visualization.

## 3. Results

### 3.1. Socio-Demographic and Medical Characteristics

**Figure 2:**
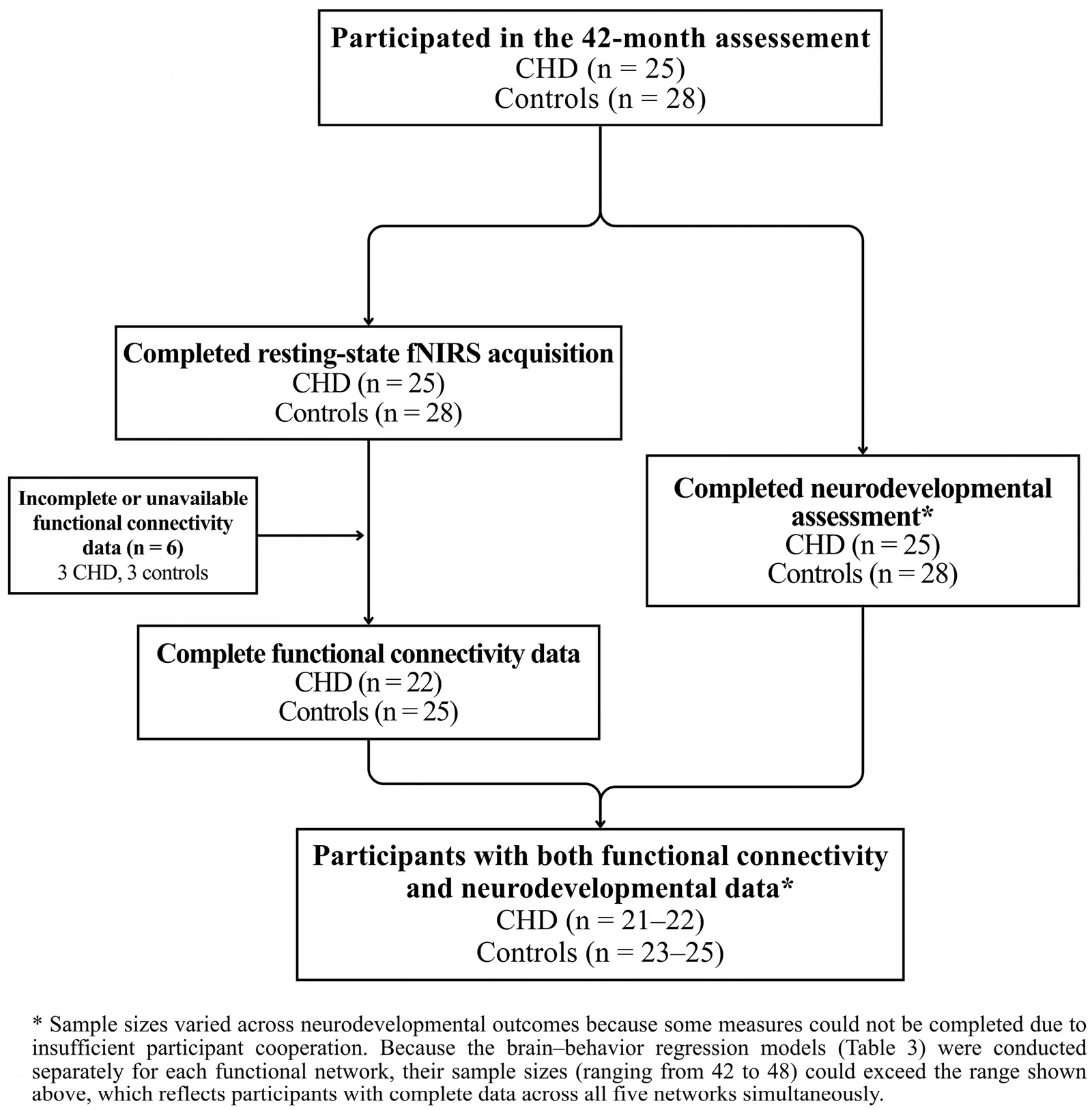
Participant flow diagram for the 42-month assessment. The diagram summarizes participant completion of the resting-state fNIRS and neurodevelopmental assessments and the availability of data for functional connectivity and brain–behavior analyses. Sample sizes varied across neurodevelopmental outcomes because some measures could not be completed due to insufficient participant cooperation.

Participant flow through the 42-month assessment and the availability of resting-state fNIRS and neurodevelopmental data are presented in Figure 2. Medical and sociodemographic characteristics are presented in Table 1. A chi-square test of independence revealed a significant difference in sex distribution between groups: χ^2^(1) = 8.11, and *p* = 0.004. The control group included a higher proportion of girls (53.6%; 15/28) than the CHD group (12%; 3/25). Age at neurodevelopmental assessment (CHD: 43.77 months; controls: 43.33 months) and at fNIRS recording (CHD: 44.06 months; controls: 43.41 months) did not differ significantly between groups. No significant group differences were observed in maternal socioeconomic status. Children with CHD had significantly lower Apgar scores at 5 min than controls (median [range]: 8 [4,5,6,7,8,9,10] vs. 9 [8,9,10]; Mann–Whitney U = 217.50, *p* = 0.022).

### 3.2. Resting-State Functional Brain Connectivity

The repeated-measures ANCOVA included 46 participants (21 CHD and 25 controls) with complete connectivity and covariate data. After preprocessing, all participants in the full fNIRS sample had at least 7 min of usable recording, and no recordings were excluded. No significant group differences were observed in any of the fNIRS quality-control metrics. Quality-control metrics are reported in Table S2.

**Figure 3:**
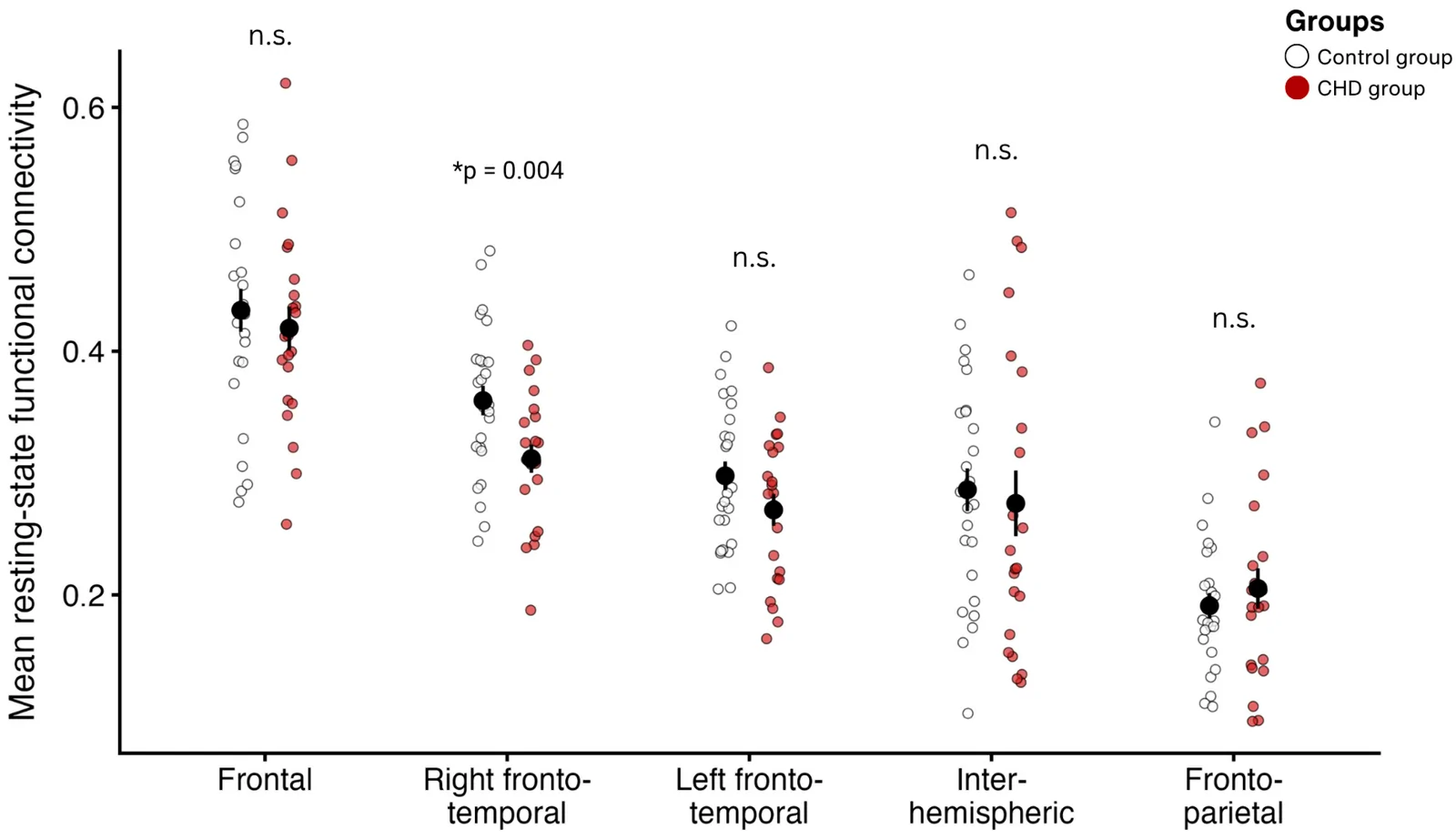
Mean resting-state functional connectivity by functional network and group. Networks are ordered by mean connectivity strength. Individual points represent participants (white circles: controls; red circles: children with CHD). Black circles indicate group means with error bars representing ±1 standard error. * *p* = 0.004, Bonferroni-corrected across the five networks; no group differences were observed in the remaining four networks (non-significant, *p* > 0.05) (n.s.).

Mauchly’s test indicated a violation of the sphericity assumption, W = 0.643, χ^2^(9) = 17.42, *p* = 0.043; Greenhouse-Geisser and Huynh-Feldt corrections yielded the same conclusions as the uncorrected tests. A main effect of network was observed, F(3.15, 129.10) = 6.11, *p* < 0.001, ηp^2^ = 0.130, indicating that mean functional connectivity differed among the five networks, regardless of group (Figure 3). Bonferroni-adjusted post hoc comparisons showed that the frontal network exhibited significantly greater functional connectivity than all other networks (all *p* < 0.001; mean differences ranging from 0.101 to 0.239), whereas the fronto-parietal network showed significantly lower functional connectivity than all other networks (all *p* ≤ 0.004; mean differences ranging from −0.239 to −0.077). The right fronto-temporal network showed greater connectivity than both the left fronto-temporal (*p* = 0.007) and inter-hemispheric networks (*p* = 0.040), which did not differ from each other (*p* = 1.000).

Group differences were then examined within each network separately. Children with CHD showed significantly lower functional connectivity than controls in the right fronto-temporal network, F(1, 42) = 9.48, *p* = 0.004, ηp^2^ = 0.184 (estimated marginal means: CHD = 0.309, 95% CI [0.281, 0.338]; controls = 0.366, 95% CI [0.343, 0.389]). No group differences were observed in the left fronto-temporal (F(1, 42) = 1.22, *p* = 0.275, ηp^2^ = 0.028), frontal (F(1, 42) = 0.02, *p* = 0.892, ηp^2^ < 0.001), inter-hemispheric (F(1, 42) = 0.06, *p* = 0.816, ηp^2^ = 0.001), or fronto-parietal networks (F(1, 42) = 0.38, *p* = 0.539, ηp^2^ = 0.009) (Figure 3).

In the repeated-measures model, no main effect of group was observed, F(1, 41) = 1.48, *p* = 0.230, ηp^2^ = 0.035, consistent with the localized rather than diffuse nature of the group difference. No main effect of sex was observed (F(1, 41) = 0.05, *p* = 0.824, ηp^2^ = 0.001), and neither the group × sex (F(1, 41) = 0.87, *p* = 0.355, ηp^2^ = 0.021) nor the network × group interaction reached significance (F(3.15, 129.10) = 1.68, *p* = 0.171, ηp^2^ = 0.039). To further assess the potential influence of the sex imbalance between groups, a sensitivity analysis restricted to male participants was conducted. The main effect of group (F(1, 24) = 0.15, *p* = 0.700, ηp^2^ = 0.006) and the network × group interaction (F(4, 96) = 0.74, *p* = 0.564, ηp^2^ = 0.030) remained non-significant in this subsample, consistent with the full-sample pattern of a network-specific rather than global alteration. The group difference in right fronto-temporal connectivity remained significant (F(1, 24) = 4.54, *p* = 0.044, ηp^2^ = 0.159), with a comparable effect size to that observed in the full sample, suggesting that the observed right fronto-temporal group difference was not solely attributable to the sex imbalance between groups. A sensitivity analysis using HbR-derived connectivity confirmed this pattern, with a significant main effect of network (F(3.15, 103.82) = 8.24, *p* < 0.001) but no significant effect of group (F(1, 33) = 0.03, *p* = 0.854), either as a main effect or in interaction with ROI (all *p*s > 0.52).

### 3.3. Neurodevelopmental Results

No group differences were observed after correction for multiple comparisons in the cognitive or motor domains (Table 2; raw *p*-values in Table S1).

**Figure 4:**
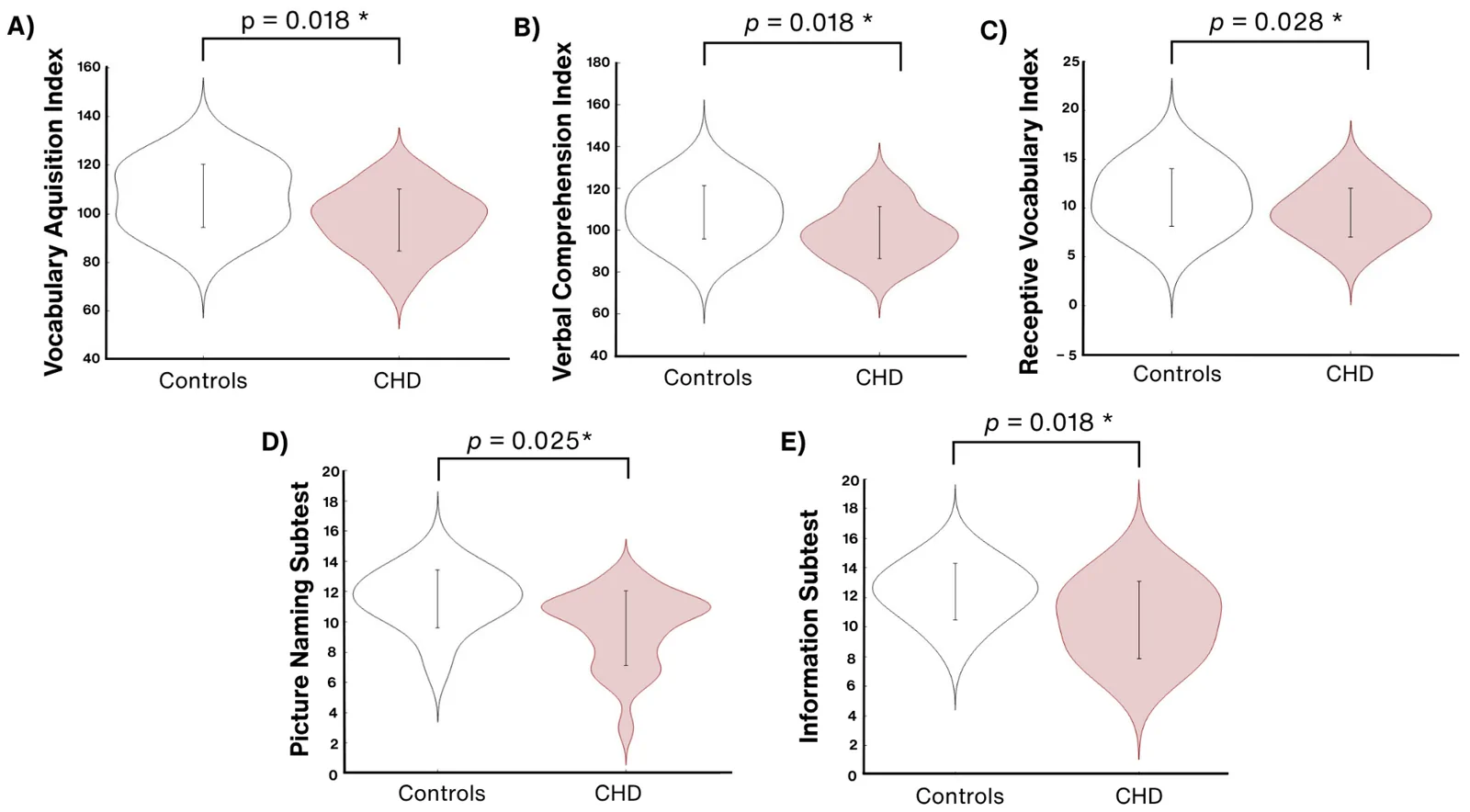
Violin plots of language outcomes in children with moderate-to-severe CHD and controls. (**A**) Vocabulary Acquisition Index (VAI), (**B**) Verbal Comprehension Index (VCI), (**C**) Receptive Vocabulary subtest, (**D**) Picture Naming subtest, and (**E**) Information subtest. Error bars indicate the mean ± 1 SD. *p*-values correspond to FDR-corrected results from ANCOVA models including group and sex as factors and maternal SES as a covariate. * *p* < 0.05.

Analyses of WPPSI-IV outcomes included 50 participants (23 CHD and 27 controls) with complete SES data. Children with moderate-to-severe CHD showed lower performance in the language domain. Significant group differences were observed for the Verbal Comprehension Index (VCI; F(1,45) = 7.00, *p* = 0.018, ηp^2^ = 0.135) and the Vocabulary Acquisition Index (VAI; F(1,45) = 8.34, *p* = 0.018, ηp^2^ = 0.156). At the subtest level, children with moderate-to-severe CHD also performed more poorly on the Receptive Vocabulary subtest (F(1, 45) = 5.14, *p* = 0.028, ηp^2^ = 0.102), Information subtest (F(1, 45) = 6.13, *p* = 0.025, ηp^2^ = 0.115) and the Picture Naming subtest (F(1,45) = 7.40, *p* = 0.018, ηp^2^ = 0.141) (Figure 4).

### 3.4. Associations Between Resting-State Functional Connectivity and Neurodevelopmental Results

**Figure 5:**
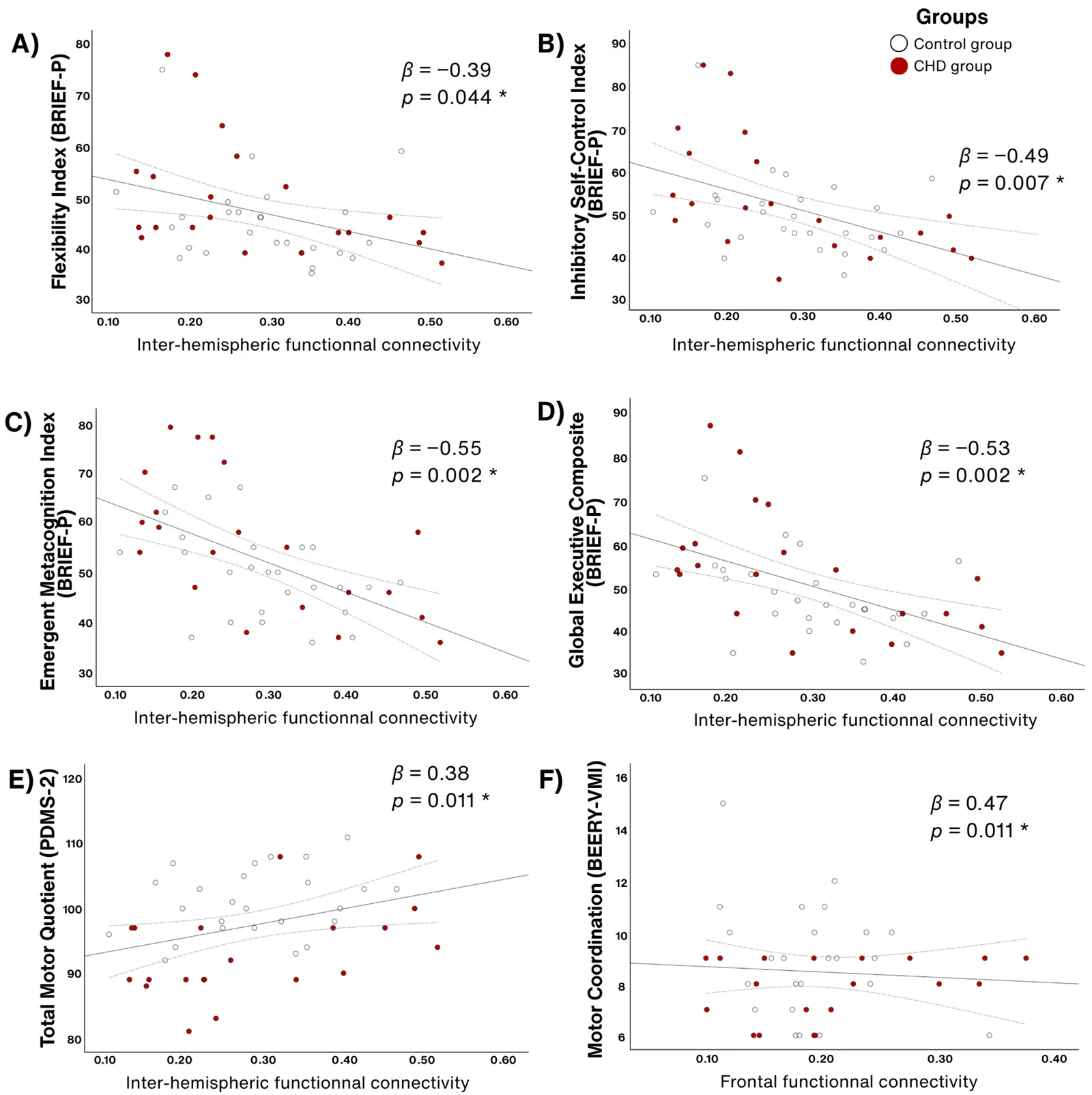
Significant associations between resting-state functional connectivity and neurodevelopmental outcomes. Scatterplots illustrate the associations between inter-hemispheric functional connectivity and BRIEF-P scores for the (**A**) Flexibility Index (FI), (**B**) Inhibitory Self-Control Index (ISCI), (**C**) Emergent Metacognition Index (EMI), and (**D**) Global Executive Composite (GEC); between inter-hemispheric connectivity and (**E**) the PDMS-2 Total Motor Quotient (TMQ); and between frontal connectivity and (**F**) Beery-VMI Motor Coordination (MC) across all participants. White circles represent controls and red circles children with CHD; solid lines indicate the linear fit across all participants, with 95% confidence bands. Higher BRIEF-P scores indicate greater executive function difficulties, whereas higher PDMS-2 and Beery scores indicate better motor performance. Fitted lines are based on unadjusted values, whereas the reported analyses control for diagnostic group, sex, and socioeconomic status. * *p* < 0.05.

Because each regression model required complete data only for the specific functional network and neurodevelopmental measure being tested, sample sizes varied across the brain–behavior regression models, ranging from 42 to 46 participants total. Hierarchical regression analyses first examined associations between resting-state functional connectivity and neurodevelopmental outcomes after adjusting for diagnostic group, sex, and socioeconomic status. After FDR correction, greater inter-hemispheric functional connectivity was significantly associated with fewer executive function difficulties, as reflected by lower scores on the BRIEF-P Global Executive Composite (ΔR^2^ = 0.277, *p* = 0.002), Emergent Metacognition Index (ΔR^2^ = 0.303, *p* = 0.002), Inhibitory Self-Control Index (ΔR^2^ = 0.243, *p* = 0.007), and Flexibility Index (ΔR^2^ = 0.155, *p* = 0.044). Greater inter-hemispheric connectivity was also associated with better motor performance on the PDMS-2 Total score (ΔR^2^ = 0.147, *p* = 0.011), and greater frontal connectivity with better Beery Motor Coordination performance (ΔR^2^ = 0.209, *p* = 0.011) (see Figure 5; Table 3). No other connectivity–neurodevelopmental associations were found. Connectivity-by-group interactions were subsequently examined to determine whether these associations differed between children with CHD and controls. No significant connectivity-by-group interactions were observed, indicating that these associations did not significantly differ between groups.

## 4. Discussion

The aim of this study was to characterize resting-state functional connectivity alterations in preschool-aged children with moderate-to-severe CHD and to examine whether these alterations were associated with neurodevelopmental functioning in the cognitive, language, and motor domains. Overall, our findings suggest that preschool-aged children with moderate-to-severe CHD exhibit lower resting-state functional connectivity within the right fronto-temporal network as well as poorer language abilities compared with typically developing peers. Greater inter-hemispheric connectivity was associated with fewer executive function difficulties and better motor performance across participants, and greater frontal connectivity with better motor coordination, with no evidence that these associations differed between children with CHD and controls. Together, these findings suggest that connectivity differences are detectable during the preschool years in children with CHD, and that they may be regionally circumscribed rather than widespread.

### 4.1. Resting-State Brain Functional Connectivity Alterations

Children with moderate-to-severe CHD demonstrated weaker resting-state functional connectivity than controls specifically in the right fronto-temporal network, with no group differences emerging in the other networks, suggesting a more circumscribed rather than a globally distributed difference in functional brain organization. Similar alterations have been reported in infants with CHD both before and after cardiac surgery [25,30], as well as in adolescents and adults [28]. This suggests that differences in functional brain organization may be detectable across multiple developmental periods in individuals with CHD. These differences could be related to structural brain abnormalities reported in children with CHD, including white matter injury, reduced cortical volumes, altered myelination and gyrification, and developmentally immature structural connectomes [21,27,69]. Prior work has linked early structural brain immaturity, particularly involving white matter microstructure, to subsequent alterations in functional connectivity [35], suggesting a plausible link between atypical structural and functional brain development. Because large-scale functional networks begin to emerge prenatally, it is possible that early cerebral disturbances associated with CHD concurrently affect structural and functional brain development, which could contribute to the functional connectivity alterations observed here [28,35,70]. However, in the absence of structural or diffusion imaging data in the present cohort, this remains a biologically plausible hypothesis rather than a conclusion supported by the current findings.

Given the rapid maturation of functional networks during the preschool years [55,56,71], we next examined the neurodevelopmental profiles of our cohort and whether resting-state functional connectivity was associated with neurodevelopmental outcomes.

### 4.2. Neurodevelopmental Vulnerabilities in Preschool Children with CHD

More specifically, children with CHD obtained lower scores on the Receptive Vocabulary (receptive vocabulary), Picture Naming (expressive vocabulary), and Information (acquired knowledge) subtests, as well as on the Vocabulary Acquisition Index (VAI) and Verbal Comprehension Index (VCI), suggesting weaker language abilities across both expressive and receptive domains. This pattern is consistent with previous reports of early language deficits in CHD [32,72]. Given that early language abilities predict later academic and adaptive outcomes, the language difficulties observed here may carry long-term implications consistent with those previously reported in CHD [73,74].

Beyond language, no group differences were observed in cognitive, executive, or motor functioning. Although previous studies have reported difficulties in these domains among children with CHD [7,32,75,76], findings remain inconsistent, possibly reflecting the subtlety of early impairments [72,77]. In addition, the BRIEF-P, as a parent-reported measure, may be less sensitive than performance-based assessments for detecting subtle executive difficulties [78]. Similarly, the absence of group differences in motor functioning may reflect the rapid motor development that characterizes the preschool years [72,77]. Neurodevelopmental outcomes in children with CHD are also highly heterogeneous, which may further reduce the ability to detect consistent group differences across domains [79]. Finally, all children with CHD in our cohort were followed through the “Clinique d’investigation neurocardiaque” (CINC) at CHU Sainte-Justine, which provides systematic developmental screening and access to early intervention services for children with moderate-to-severe CHD, including physical and occupational therapies. In addition, some children may have received developmental or therapeutic services outside the hospital setting; however, information regarding these services was not available in hospital records for several participants. Such interventions may have supported developmental outcomes and attenuated group differences at preschool age [80].

### 4.3. Resting-State Functional Connectivity and Neurodevelopmental Outcomes

We also examined whether resting-state functional connectivity was associated with neurodevelopmental outcomes. Greater inter-hemispheric functional connectivity was associated with fewer parent-reported executive difficulties on the BRIEF-P across all participants, including lower scores on the Global Executive Composite and its constituent indices of Inhibitory Self-Control, Flexibility, and Emergent Metacognition. Because the GEC is derived from its constituent indices, these associations are not statistically independent and should be interpreted as converging evidence for an association between inter-hemispheric connectivity and executive functioning rather than as four independent findings. Similar associations between functional connectivity and both performance-based and parent-reported measures of executive functioning have been reported in typically developing children from infancy through later childhood [34,81,82]. Importantly, these associations remained significant after controlling for diagnostic group, sex, and maternal SES and after correction for multiple comparisons, suggesting that inter-hemispheric functional connectivity was associated with executive functioning beyond differences attributable to these factors.

This association may reflect the importance of large-scale network integration for executive functioning during early development. Although executive functions rely strongly on frontal and parietal regions, they emerge from coordinated activity across distributed cortical networks involved in cognitive control, monitoring, and information integration [25,83,84,85,86]. These networks undergo substantial maturation during early childhood, with increasing functional integration supporting the development of more efficient executive processes [37,81,87]. Inter-hemispheric connectivity may therefore capture an aspect of distributed network integration relevant to emerging executive abilities.

We also found that functional connectivity was associated with motor outcomes. More specifically, greater inter-hemispheric connectivity was associated with better overall motor performance on the PDMS-2 Total Motor Quotient, and greater frontal connectivity was associated with better Motor Coordination on the Beery-VMI Motor Coordination subtest. This could be explained by the fact that the PDMS-2 total motor quotient takes into account both fine and gross motor skills, and may be supported by inter-hemispheric integration across frontal, parietal, and temporal association circuitries, which supports increasingly adaptive and efficient motor behaviors with age [88]. In contrast, the Beery-VMI Motor Coordination task is based solely on one-handed drawing within set boundaries, which may require less whole-brain coordination and rely more on primary motor areas for simple, goal-directed voluntary movement [89].

No significant connectivity-by-group interactions were observed for any of these associations, indicating that they did not significantly differ between children with CHD and controls. This suggests that the relationships between inter-hemispheric connectivity and executive and motor functioning, as well as between frontal connectivity and motor coordination, may be similar across groups, despite differences in overall functional connectivity between children with CHD and controls. Inter-hemispheric connections emerge early and become increasingly integrated across homotopic regions over development [37], while frontal connectivity continues to mature during early childhood [56], suggesting that these brain–behavior relationships may reflect a broader developmental feature of functional network organization rather than one specific to CHD. However, given the modest sample size, smaller group differences in these associations cannot be excluded.

### 4.4. Limitations

This study has several limitations that should be considered when interpreting the findings. First, the sample size was relatively small, particularly for a neuroimaging study, which may have limited our ability to detect more subtle group differences and brain-behavior associations. A power analysis using G*Power (version 3.1.9.6) indicated that the present sample was adequately powered to detect large effects but not small or medium effects. Recruiting large samples remains particularly challenging in pediatric CHD research because of the rarity and clinical heterogeneity of this population. This is even more difficult in early childhood when many children still have regular medical follow-up appointments and undergo cardiac surgery.

Second, the unequal distribution of males and females across groups may have influenced the results, especially given the significant effects of sex observed for several neurodevelopmental measures. Including sex as a factor in our statistical analyses allowed us to estimate the unique effect of group while simultaneously accounting for the effect of sex and its potential interaction [90]; nevertheless, the imbalance remains a potential source of bias. In the sensitivity analysis restricted to male participants, the group difference in right fronto-temporal connectivity remained significant, with a comparable effect size to that observed in the full sample, indicating that this finding was not driven by the sex imbalance between groups. Neither the main group effect nor the network × group interaction reached statistical significance, consistent with the full-sample pattern of a network-specific rather than global alteration. Moreover, the very small number of girls in the CHD group precluded a reliable assessment of sex-specific effects. Although this imbalance is consistent with the greater prevalence of complex CHD among males [91], it does not eliminate the potential influence of sex on the findings. Replication in larger, more sex-balanced samples is therefore needed.

Third, a sensitivity analysis using HbR-derived connectivity did not show a significant group effect, suggesting that the right fronto-temporal difference observed with HbO does not extend to HbR. This divergence is not unexpected, as HbR is known to yield a lower signal-to-noise ratio than HbO [52], and the relatively small sample size of this sensitivity analysis further limits its power to detect group differences.

Additionally, although eight short-separation detectors were included in the acquisition montage to account for extracerebral signal contamination, short-separation regression was not applied in the final preprocessing pipeline, as it appeared to remove signal of interest (i.e., cerebral signal) rather than isolating superficial physiological noise. One possible explanation is that the 8-mm source–detector separation may not have been optimal for preschool-aged children given developmental anatomical differences with older children or adults, which may have affected the extent to which short-separation channels captured extracerebral versus cerebral signal [47]. This represents a methodological limitation, and future studies should consider age-appropriate short-separation distances and optimized approaches for handling systemic physiological confounds in fNIRS connectivity analyses. To assess whether reliance on GSR in place of short-separation regression influenced our findings, we repeated the primary analysis without GSR; results were consistent with the GSR-corrected analysis and are reported as a Supplementary Result.

Furthermore, the cross-sectional nature of the present study does not allow conclusions regarding the developmental trajectories of functional connectivity alterations or their causal relationship with neurodevelopmental outcomes. Longitudinal studies are needed to determine whether early alterations in brain network organization predict later developmental difficulties.

Finally, CHD encompasses a heterogeneous range of cardiac malformations. Although participants were recruited within a similar level of cardiac severity, the CHD group was not composed of a single diagnostic category. However, nearly all children in our cohort had biventricular physiology, which may limit the generalizability of our findings to children with univentricular physiology, who are generally at greater risk for adverse neurodevelopmental outcomes [92]. While this supports the generalizability of our results across different CHD diagnoses with predominantly biventricular physiology, differences in cardiac diagnosis, medical history, and perioperative factors may contribute to variability in both neurodevelopmental outcomes and functional brain organization.

## 5. Clinical Implications and Future Directions

This study is among the first to examine resting-state functional connectivity and its relationship with neurodevelopmental functioning in preschool-aged children with CHD. Children with CHD showed lower resting-state functional connectivity within the right fronto-temporal network and poorer language performance than typically developing peers. Greater inter-hemispheric connectivity was associated with fewer parent-reported executive function difficulties and better motor performance, and greater frontal connectivity was associated with better motor coordination. These associations did not differ significantly between children with CHD and controls, although the modest sample size limits the detection of smaller group differences. Collectively, these findings indicate that alterations in functional brain organization are detectable at preschool age in children with CHD and that they are regionally circumscribed rather than global. They also point to an association between inter-hemispheric and frontal functional integration and both executive and motor functioning that was comparable across groups and that may therefore reflect a broader feature of early network development rather than a CHD-specific mechanism.

Given the high degree of brain plasticity during early childhood [71], this developmental period represents a critical window for identifying children at risk and implementing early interventions. Longitudinal studies integrating neuroimaging and developmental assessments are needed to clarify the developmental significance of atypical connectivity patterns, determine whether early differences in functional brain organization are associated with subsequent neurodevelopmental trajectories, and evaluate their potential as a candidate research marker of neurodevelopmental outcomes in children with CHD.

## Figures and Tables

**Table 1:** Participant and medical characteristics.

	Controls (*n* = 28)	CHD (*n* = 25)	*p*	Skewness/ Kurtosis
Female sex, *n* (%)	15 (53.57)	3 (12)	0.004 *	-
Age at neurodevelopmental assessment (months)	43.3 (1.64)	43.8 (1.50)	0.342	0.86/0.5
Age at fNIRS recording (months)	43.4 (1.59)	44.1 (1.57)	0.108	0.80/0.40
Maternal socioeconomic status *^a^*	77.45 (21.46)	74.26 (19.46)	0.623	−1.58/2.79
Antenatal CHD diagnosis, *n* (%)	-	21 (84.0)	-	-
Apgar score at 5 min, median (range)	9 (8–10)	8 (4–10)	0.022 *	-
Age at first surgery, days, median (IQR)	-	9 (7–15)	-	-
Number of cardiac surgeries, *n* (%)	-		-	-
1	-	21 (84.0)	-	-
2	-	4 (16.0)	-	-
Length of PICU stay, days, median (IQR)	-	6 (4–8)	-	-
Total hospitalization duration, days, median (IQR)	-	18 (13–21)	-	-
Cyanotic status, *n* (%)	-		-	-
Cyanotic	-	20 (80.0)	-	-
Acyanotic	-	5 (20.0)	-	-
Cardiopulmonary bypass duration, min, mean (SD)	-	150 (76)	-	-
Aortic cross-clamp duration, min, mean (SD)	-	107 (49)	-	-
Ventricular physiology, *n* (%)	-			
Biventricular		24 (96.0)		
Univentricular		1 (4.0)		
Primary cardiac diagnosis, *n* (%)	-		-	-
Transposition of the great arteries	-	14 (56.0)	-	-
Tetralogy of Fallot	-	2 (8.0)	-	-
Double outlet right ventricle	-	3 (12.0)	-	-
Coarctation of the aorta	-	2 (8.0)	-	-
Truncus arteriosus	-	1 (4.0)	-	-
Total anomalous pulmonary venous return	-	1 (4.0)	-	-
Complete atrioventricular septal defect	-	1 (4.0)	-	-
Hypoplastic aortic arch	-	1 (4.0)	-	-

Data are presented as mean (standard deviation (SD)) for normally distributed continuous variables, median (interquartile range (IQR) or range, as indicated) for non-normally distributed continuous variables, and *n* (%) for categorical variables. CHD, congenital heart disease; PICU, pediatric intensive care unit. * *p* < 0.05. Higher socioeconomic status scores indicate higher social status. *^a^* Higher socioeconomic status scores indicate higher social status.

**Table 2:** Neurodevelopmental results by domain.

	Controls (*n* = 27)	CHD (*n* = 23)	*p*
Cognitive Domain			
WPPSI-IV			
General ability index (GAI)	111.86 (12.69)	101.51 (9.96)	0.056
Visual spatial index (VSI)	109.42 (11.85)	104.22 (10.18)	0.289
Block design	11.43 (2.22)	10.20 (2.17)	0.565
Object assembly	11.85 (2.63)	10.23 (2.18)	0.289
BRIEF-P			
Global Executive Composite (GEC)	47.93 (9.05)	54.65 (15.00)	0.289
Inhibitory Self-Control Index (ISCI)	49.46 (9.76)	53.12 (14.41)	0.492
Flexibility Index (FI)	45.86 (8.38)	52.10 (13.99)	0.289
Emergent Metacognition Index (EMI)	49.15 (8.93)	55.62 (13.54)	0.289
Language Domain			
WPPSI-IV			
Verbal comprehension index (VCI)	109.61 (12.78)	98.52 (12.41)	0.018 *
Vocabulary acquisition index (VAI)	109.23 (12.98)	96.32 (12.66)	0.018 *
Receptive vocabulary	11.27 (2.82)	9.52 (2.50)	0.028 *
Information	12.54 (1.93)	10.28 (2.60)	0.025 *
Picture naming	11.97 (1.92)	9.24 (2.47)	0.018 *
Motor Domain			
Beery-VMI			
Visuo-motor integration (VMI)	11.16 (1.30)	11.48 (1.24)	0.444
Motor coordination (MC)	8.96 (2.09)	8.43 (1.26)	0.444
PDMS-2			
Total motor quotient (TMQ)	100.80 (5.06)	95.20 (7.26)	0.063
Fine motor quotient (FMQ)	109.99 (11.20)	100.96 (9.78)	0.397
Gross motor quotient (GMQ)	95.11 (4.95)	91.93 (6.95)	0.063

Data are presented as mean (SD). *p*-values were calculated using analysis of covariance (ANCOVA) models including group and sex as factors and socioeconomic status (SES) as a covariate. *p*-values are adjusted for multiple comparisons using the Benjamini–Hochberg false discovery rate (FDR) procedure, applied separately within each neurodevelopmental domain (cognitive, language, motor). * Adjusted *p* < 0.05. Raw *p*-values are provided in Table S1. WPPSI-IV, Wechsler Preschool and Primary Scale of Intelligence–Fourth Edition; BRIEF-P, Behavior Rating Inventory of Executive Function–Preschool Version; Beery-VMI, Beery–Buktenica Developmental Test of Visual–Motor Integration; PDMS-2, Peabody Developmental Motor Scales–Second Edition.

**Table 3:** Significant associations between resting-state functional connectivity and neurodevelopmental results.

		Network	β	ΔR^2^	*pFDR*
Cognitive Domain					
	BRIEF-P				
	Global Executive Composite (GEC)	Inter-hemispheric	−0.53	0.277	0.002
	Flexibility Index (FI)	Inter-hemispheric	−0.39	0.155	0.044
	Emergent Metacognition Index (EMI)	Inter-hemispheric	−0.55	0.303	0.002
	Inhibitory Self-Control Index (ISCI)	Inter-hemispheric	−0.49	0.243	0.007
Motor Domain					
	Beery-VMI				
	Motor Coordination (MC)	Frontal	0.47	0.209	0.011
	PDMS-2				
	Total Motor Quotient (TMQ)	Inter-hemispheric	0.38	0.147	0.011

β = standardized coefficient for connectivity entered at step 2 of the hierarchical regression, adjusted for diagnostic group, sex, and maternal SES. ΔR^2^ = increment in explained variance attributable to connectivity. *p* = false discovery rate–corrected *p*-value within neurodevelopmental domain. Higher BRIEF-P scores indicate greater executive function difficulties; higher PDMS-2 and Beery scores indicate better performance. Only associations surviving FDR correction are shown; full results are reported in Supplementary Table S3.

## Data Availability

The data used for this study are confidential.

## Supplementary Materials

The following supporting information can be downloaded at https://www.mdpi.com/article/10.3390/biomedicines14102226/s1. Table S1: Raw *p*-values for neurodevelopmental domain comparisons; Table S2: fNIRS quality-control metrics; Table S3: Associations between resting-state functional connectivity and neurodevelopmental outcomes; and Supplementary Result: Sensitivity analysis without global signal regression.

## Abbreviations

The following abbreviations are used in this manuscript:

CHD	Congenital Heart Disease
CINC	Clinique d’investigation neurocardiaque
fNIRS	Functional Near-Infrared Spectroscopy
SES	Socioeconomic Status
PCA	Principal Component Analysis
PARAFAC	Parallel Factor Analysis
WPPSI-IV	Wechsler Preschool and Primary Scale of Intelligence, Fourth Edition
VCI	Verbal Comprehension Index
VSI	Visual Spatial Index
VAI	Vocabulary Acquisition Index
GAI	General Ability Index
BRIEF-P	Behavior Rating Inventory of Executive Function, preschool version
ISCI	Inhibitory Self-Control Index
FI	Flexibility Index
EMI	Emergent Metacognition Index
GEC	Global Executive Composite
PDMS-2	Peabody Developmental Motor Scales, second edition
FMQ	Fine Motor Quotient
GMQ	Gross Motor Quotient
TMQ	Total Motor Quotient
BEERY-VMI	Beery–Buktenica Developmental Test of Visual–Motor Integration
MC	Motor Coordination
VMI	Visual–Motor Integration
ANCOVA	Analysis of Covariance
HbO	Oxyhemoglobin
HbR	Deoxyhemoglobin
ROI	Region of Interest
FDR	False Discovery Rate
GSR	Global Signal Regression
PICU	Pediatric Intensive Care Unit
IQR	Interquartile Range
