# Supplementary material for: Resting-State Functional Brain Connectivity and Neurodevelopmental Profiles in 3-Year-Olds with Congenital Heart Disease
Source: Biomedicines. 2026 Oct 1;14(10):2226. doi: 10.3390/biomedicines14102226 (PMC13701384; doi:10.3390/biomedicines14102226)
Supplement: Supplementary file 1 [file biomedicines-14-02226-s001.zip › biomedicines-4502754-supplementary.pdf]

Supplementary Tables

Table S1. Raw p-values for neurodevelopmental domain comparisons (corresponding to Table 2).

	Raw p-values
Cognitive Domain	
WPPSI-IV	
General ability index (GAI)	0.007
Visual spatial index (VSI)	0.182
Block design	0.565
Object assembly	0.119
BRIEF-P	
Global Executive Compo site (GEC)	0.166
Inhibitory Self-Control Index (ISCI)	0.431
Flexibility Index (FI)	0.089
Emergent Metacognition Index (EMI)	0.217
Language Domain	
WPPSI-IV	
Verbal comprehension index (VCI)	0.011
Vocabulary acquisition index (VAI)	0.006
Receptive vocabulary	0.028
Information	0.020
Picture naming	0.009
Motor Domain	
Beery-VMI	
Visuo-motor integration (VMI)	0.444
Motor coordination (MC)	0.423
PDMS-2	
Total motor quotient (TMQ)	0.019
Fine motor quotient (FMQ)	0.238
Gross motor quotient (GMQ)	0.025

Table S2. fNIRS quality-control metrics

	Controls (n = 28)	CHD (n = 25)	t-test
Average recording duration (s)	652 ± 107	724 ± 160	p = 0.063
Number of rejected channels	19.6 ± 18.0	15.6 ± 11.5	p = 0.367
Percentage of rejected channels (%)	26.8 ± 24.7	21.3 ± 15.8	p = 0.367
Number of random trials used for connectivity	159 ± 34.5	166 ± 28.6	p = 0.439
Number of corrected motion-contaminated segments	14.1 ± 5.0	13.1 ± 6.5	p = 0.555
Number of components removed	69.04 ± 42.43	84.81 ± 69.53	p = 0.325

Table S3. Associations between resting-state functional connectivity and neurodevelopmental outcomes

		Left fronto-temporal		Right fronto-temporal		Frontal		Inter-hemispheric		Fronto-parietal	
		Step 2	*Step 3*	*Step 2*	*Step 3*	*Step 2*	*Step 3*	*Step 2*	*Step 3*	*Step 2*	*Step 3*
Cognitive Domain											
WPPSI-IV											
GAI	*β*	.12	.18	.23	.19	.11	.03	.08	.10	.02	.62
	p	.337	.305	.094	.275	.395	.833	.544	.615	.891	.011
	pFDR	.665	.961	.313	.961	.688	.961	.803	.961	.963	.456
VSI	*β*	.01	.38	.21	.07	.08	.01	.04	.02	-.03	.60
	p	.920	.058	.186	.730	.592	.939	.766	.926	.841	.035
	pFDR	.968	.707	.460	.961	.803	.961	.875	.961	.934	.706
Block design	*β*	-.08	.35	.21	.18	.16	-.04	.15	.17	.07	.49
	p	.615	.093	.195	.391	.284	.825	.293	.495	.653	.093
	pFDR	.803	.707	.460	.961	.618	.961	.618	.961	.803	.707
Object assembly	*β*	.09	.26	.15	-.06	-.01	.04	-.06	-.12	-.11	.46
	p	.558	.198	.366	.762	.960	.844	.663	.605	.448	.106
	pFDR	.803	.961	.665	.961	.984	.961	.803	.961	.747	.707
BRIEF-P											
GEC	*β*	.20	-.02	-.36	.13	-.37	-.15	**-.53**	-.08	-.07	-.07
	p	.186	.934	.030	.524	.012	.428	**<.001 ***	.695	.647	.829
	pFDR	.460	.961	.149	.961	.083	.961	**.002 ***	.961	.803	.961
ISCI	*β*	.11	-.01	-.24	.01	-.34	-.24	**-.49**	-.18	-.00	.02
	p	.485	.961	.166	.960	.026	.218	**<.001 ***	.407	.998	.947
	pFDR	.776	.961	.460	.961	.149	.961	**.007 ***	.961	.998	.961
FI	*β*	.14	.03	-.32	.19	-.29	-.14	**-.39**	-.01	-.08	-.03
	p	.354	.873	.045	.339	.048	.448	**.004 ***	.959	.611	.913
	pFDR	.665	.961	.191	.961	.191	.961	**.044 ***	.961	.803	.961
EMI	*β*	.22	-.05	-.32	.13	-.39	-.09	**-.55**	-.12	-.05	-.10
	p	.152	.807	.057	.550	.011	.628	**<.001 ***	.567	.743	.744
	pFDR	.460	.961	.208	.961	.083	.961	**.002 ***	.961	.874	.961
Language Domain											
WPPSI-IV											
VAI	*β*	.13	.19	.29	.22	.06	.12	.07	.26	.06	.62
	p	.308	.296	.037	.190	.670	.495	.600	.211	.663	.013
	pFDR	.628	.548	.425	.513	.797	.681	.789	.513	.797	.262
VCI	*β*	.19	-.02	.24	.22	.09	.09	.09	.20	.08	.51
	p	.153	.896	.088	.215	.499	.602	.482	.345	.528	.046
	pFDR	.425	.974	.425	.513	.733	.717	.733	.575	.733	.262
Receptive vocabulary	*β*	.20	-.00	.22	.22	.04	.07	-.01	.19	-.03	.57
	p	.123	.987	.125	.216	.737	.677	.958	.392	.836	.026
	pFDR	.425	.987	.425	.513	.814	.769	.958	.605	.871	.262
Information	*β*	.13	-.00	.22	.22	.13	.11	.20	.18	.21	.31
	p	.352	.987	.151	.246	.338	.540	.130	.412	.125	.244
	pFDR	.628	.987	.425	.513	.628	.681	.425	.605	.425	.513
Picture naming	*β*	.04	.37	.27	.23	.09	.11	.16	.23	.14	.54
	p	.749	.052	.071	.203	.512	.545	.217	.307	.307	.042
	pFDR	.814	.262	.425	.513	.733	.681	.541	.548	.628	.262

		Left fronto-temporal		Right fronto-temporal		Frontal		Inter-hemispheric		Fronto-parietal	
		Step 2	*Step 3*	*Step 2*	*Step 3*	*Step 2*	*Step 3*	*Step 2*	*Step 3*	*Step 2*	*Step 3*
Motor Domain											
Beery-VMI											
VMI	*β*	-.18	.29	-.18	-.26	.26	.01	.08	.06	-.08	.01
	p	.209	.127	.247	.174	.067	.937	.566	.781	.575	.984
	pFDR	.346	.436	.346	.436	.210	.976	.599	.915	.599	.984
MC	*β*	-.25	.17	.19	-.38	**.47**	-.23	.35	-.40	.11	.22
	p	.096	.426	.270	.061	**<.001 ***	.159	.015	.072	.466	.499
	pFDR	.240	.693	.356	.299	**.011 ***	.436	.092	.299	.542	.693
PDMS-2											
TMQ	*β*	-.15	.35	.18	-.09	.24	.18	**.38**	.24	.24	.46
	p	.213	.040	.186	.599	.051	.240	**<.001 ***	.164	.051	.049
	pFDR	.346	.299	.346	.788	.210	.546	**.011 ***	.436	.210	.299
GMQ	*β*	-.11	.05	.06	.03	.18	.16	.28	.26	.18	.23
	p	.477	.805	.732	.879	.236	.422	.066	.278	.249	.452
	pFDR	.542	.915	.732	.955	.346	.693	.210	.579	.346	.693
FMQ	*β*	-.11	.40	.17	-.13	.22	.11	.32	.08	.17	.51
	p	.369	.021	.212	.432	.077	.494	.008	.662	.180	.034
	pFDR	.461	.299	.346	.693	.214	.693	.067	.828	.346	.299

Hierarchical linear regression models were conducted separately for each functional network and neurodevelopmental outcome. Step 1 included diagnostic group, sex, and socioeconomic status (SES) as covariates. Step 2 added functional connectivity to assess connectivity–neurodevelopment associations. Step 3 added the connectivity × diagnostic group interaction to determine whether these associations differed between groups. β represents standardized regression coefficients. Both raw and false discovery rate (FDR)-adjusted p values are reported. FDR correction was applied separately within each neurodevelopmental domain for connectivity effects and connectivity × diagnostic group interactions. WPPSI-IV, Wechsler Preschool and Primary Scale of Intelligence–Fourth Edition; GAI, General Ability Index; VSI, Visual Spatial Index; BRIEF-P, Behavior Rating Inventory of Executive Function–Preschool Version; GEC, Global Executive Composite; ISCI, Inhibitory Self-Control Index; FI, Flexibility Index; EMI, Emergent Metacognition Index; VAI, Vocabulary Acquisition Index; VCI, Verbal Comprehension Index; Beery-VMI, Beery-Buktenica Developmental Test of Visual-Motor Integration; VMI, Visual-Motor Integration; MC, Motor Coordination; PDMS-2, Peabody Developmental Motor Scales–Second Edition; TMQ, Total Motor Quotient; GMQ, Gross Motor Quotient; FMQ, Fine Motor Quotient

Supplementary Result

Sensitivity analysis without global signal regression

To assess whether reliance on global signal regression (GSR), used in place of short-separation regression, influenced the pattern of results, the primary repeated-measures analysis was repeated on connectivity estimates derived without GSR.

Mauchly's test indicated a violation of the sphericity assumption ($W = .593$, $\chi^2(9) = 20.61$, $p = .015$); Greenhouse-Geisser corrected degrees of freedom ($\varepsilon = .78$) are reported. The main effect of network remained significant ($F(4,164) = 4.30$, $p = .002$, $\eta p^2 = .095$), consistent with the GSR-corrected analysis ($F(4,164) = 6.11$, $p < .001$, $\eta p^2 = .130$). The main effect of group remained non-significant ($F(1,41) = 1.14$, $p = .292$, $\eta p^2 = .027$), consistent with the GSR-corrected analysis ($F(1,41) = 1.48$, $p = .230$, $\eta p^2 = .035$). The network × group interaction also remained non-significant ($F(4,164) = 0.52$, $p = .720$, $\eta p^2 = .013$), consistent with the GSR-corrected analysis ($F(4,164) = 1.68$, $p = .156$, $\eta p^2 = .039$). These results indicate that the overall pattern of findings does not depend on the use of GSR. To further examine whether the right fronto-temporal group difference specifically depended on GSR, a follow-up univariate ANCOVA was conducted on connectivity estimates derived without GSR. The group difference remained significant but was attenuated relative to the GSR-corrected analysis ($F(1,44) = 5.34$, $p = .026$, $\eta p^2 = .109$, model including GRP and SES only, $N = 47$; GSR-corrected: $F(1,42) = 9.48$, $p = .004$, $\eta p^2 = .184$, model including GRP, sex, and SES, $N = 46$). As
